# Acquired resistance to immunotherapy and chemoradiation in MYC amplified head and neck cancer

**DOI:** 10.1038/s41698-024-00606-w

**Published:** 2024-05-23

**Authors:** Thomas F. Cyberski, Alka Singh, Michael Korzinkin, Vasudha Mishra, Frank Pun, Le Shen, Claudia Wing, Xiangying Cheng, Brandon Baird, Yuxuan Miao, Moshe Elkabets, Sara Kochanny, Wenji Guo, Emma Dyer, Alexander T. Pearson, Aditya Juloori, Mark Lingen, Grayson Cole, Alex Zhavoronkov, Nishant Agrawal, Evgeny Izumchenko, Ari J. Rosenberg

**Affiliations:** 1 Pritzker School of Medicine, University of Chicago, Chicago, IL USA; 2 Department of Medicine, Section of Hematology and Oncology, University of Chicago, Chicago, IL USA; 3 Insilico Medicine, Pak Shek Kok, Hong Kong; 4 Department of Surgery, University of Chicago, Chicago, IL USA; 5 Ben May Department for Cancer Research, University of Chicago, Chicago, IL USA; 6 The Shraga Segal Department of Microbiology, Immunology, and Genetics, Ben-Gurion University, Beer Sheva, Israel; 7 Harvard T.H. Chan School of Public Health, Cambridge, MA USA; 8 Department of Radiation Oncology, University of Chicago, Chicago, IL USA; 9 Department of Pathology, University of Chicago, Chicago, IL USA

**Keywords:** Head and neck cancer, Head and neck cancer

## Abstract

The proto-oncogene MYC encodes a nuclear transcription factor that has an important role in a variety of cellular processes, such as cell cycle progression, proliferation, metabolism, adhesion, apoptosis, and therapeutic resistance. MYC amplification is consistently observed in aggressive forms of several solid malignancies and correlates with poor prognosis and distant metastases. While the tumorigenic effects of MYC in patients with head and neck squamous cell carcinoma (HNSCC) are well known, the molecular mechanisms by which the amplification of this gene may confer treatment resistance, especially to immune checkpoint inhibitors, remains under-investigated. Here we present a unique case of a patient with recurrent/metastatic (R/M) HNSCC who, despite initial response to nivolumab-based treatment, developed rapidly progressive metastatic disease after the acquisition of MYC amplification. We conducted comparative transcriptomic analysis of this patient’s tumor at baseline and upon progression to interrogate potential molecular processes through which MYC may confer resistance to immunotherapy and/or chemoradiation and used TCGA-HNSC dataset and an institutional cohort to further explore clinicopathologic features and key molecular networks associated with MYC amplification in HNSCC. This study highlights MYC amplification as a potential mechanism of immune checkpoint inhibitor resistance and suggest its use as a predictive biomarker and potential therapeutic target in R/M HNSCC.

## Introduction

Locoregionally advanced head and neck squamous cell carcinoma (HNSCC) is associated with poor 5-year survival of only 40% for non-viral mediated disease, and substantial functional morbidity with combined multimodality therapy^1–4^. Due to the poor success of systemic cytotoxic chemotherapy in treating recurrent/metastatic (R/M) HNSCC, the recent clinical focus has shifted to immunotherapy with antibodies targeting T cell inhibitory receptors that function as immune checkpoints, such as programmed death 1 (PD-1). Nonetheless, PD-1 inhibitors were reported to unleash anti-tumor immunity and achieve durable clinical responses in only 15–20% of treated patients in front-line recurrent/metastatic (R/M) setting^5–8^, with 4-year overall survival rate of about 15% overall and up to 22% among cases with a PD-L1 combined positive score (CPS) of 20 or greater^5–9^. Such disparity in treatment benefits between patients, paired with the absence of any standard effective therapies that target immunotherapy resistance, necessitates the identification of predictive biomarkers to better inform clinicians’ therapeutic decisions^3^. While it was reported that higher PD-L1 expression, assessed by the CPS, is predictive of a favorable response to immune checkpoint inhibitors (ICI), CPS remains an imperfect biomarker, with the majority of patients ultimately developing therapeutic resistance. Therefore, identification of improved biomarkers of response and resistance is of great importance, as biomarker-directed therapeutics after progression on immunotherapy (such as HRAS) suggest that determining specific mechanisms driving treatment resistance in HNSCC is key to therapeutic development^10,11^. Furthermore, little is known regarding the mechanisms of resistance to ICIs in head and neck cancer, further limiting the ability to predict non-responders and to investigate novel therapeutics targeting mechanisms of resistance to ICIs in R/M HNSCC^12,13^.

A high prevalence of alterations in the *MYC* oncogene is well documented in various solid malignancies, along with its association with aggressive disease and poor clinical outcomes^14–16^. While recurrent *MYC* gain-of-function mutations were found in certain human lymphomas^17–19^, in HNSCC, amplification appears to be the predominant genetic aberration that occurs in the *MYC* gene^20^, with an estimated prevalence of 12% in the HNSCC cohort of The Cancer Genome Atlas (TCGA), whereas mutations occur in only a small subset (1.2%) of patients^21^. *MYC* amplification is known to have broad influence on the transcriptome, driving upregulation of various mitogenic and survival signaling pathways associated with cell growth, proliferative, anti-apoptotic, and metabolic processes in ovarian, breast, and lung cancers among others^22–25^. Furthermore, *MYC* plays an important role in suppressing the host anti-tumor immune response, through various mechanisms involving modifications to the tumor microenvironment via inhibitory cytokines (e.g., TGFβ and immunomodulatory molecules such as PD-L1, CD47, and MHC I)^26–29^. While the tumorigenic effects of *MYC* are well known, the molecular mechanisms by which the mutation or amplification of this gene may confer treatment resistance in HNSCC remains under-investigated^30^. A recent case report of a patient with recurrent/metastatic (R/M) HNSCC highlighted a differential response to nivolumab in metastatic lesions secondary to the acquisition of *MYC* amplification. The *MYC* amplified lesion was resistant to the ICI while all other lesions, devoid of *MYC* amplification, responded to treatment, suggesting *MYC* amplification may play a role in ICI resistance in R/M HNSCC^31^. Supporting this suggestion, another study showed that *MYC* amplification regulates PD-L1 expression and is involved in a decreased response to ICI therapy in esophageal squamous cell carcinoma^32^.

Here we present a unique case of a patient with R/M HNSCC who, despite initial response to nivolumab-based therapy, developed rapidly progressive, metastatic disease after the acquisition of a *MYC* amplification. We conducted transcriptomic analysis of this patient’s HNSCC to identify the potential molecular processes through which *MYC* amplification may confer resistance to immunotherapy. In light of these findings, we searched our in-house genomic sequencing platform and identified seven additional patients with *MYC* amplified HNSCC along with 48 *MYC* wild-type matched controls, to further elucidate the broader clinicopathologic characteristics of *MYC*-driven disease. Finally, we performed gene expression and pathway analysis using the transcriptomic data obtained from TCGA to further explore key molecular networks associated with *MYC* amplification in HNSCC. Collectively, this work seeks to highlight MYC amplification as a potential mechanism of treatment resistance and suggest its use as a predictive biomarker and a potential therapeutic target in the treatment of R/M HNSCC^24,33,34^.

## Results

### Case presentation

**Fig. 1:**
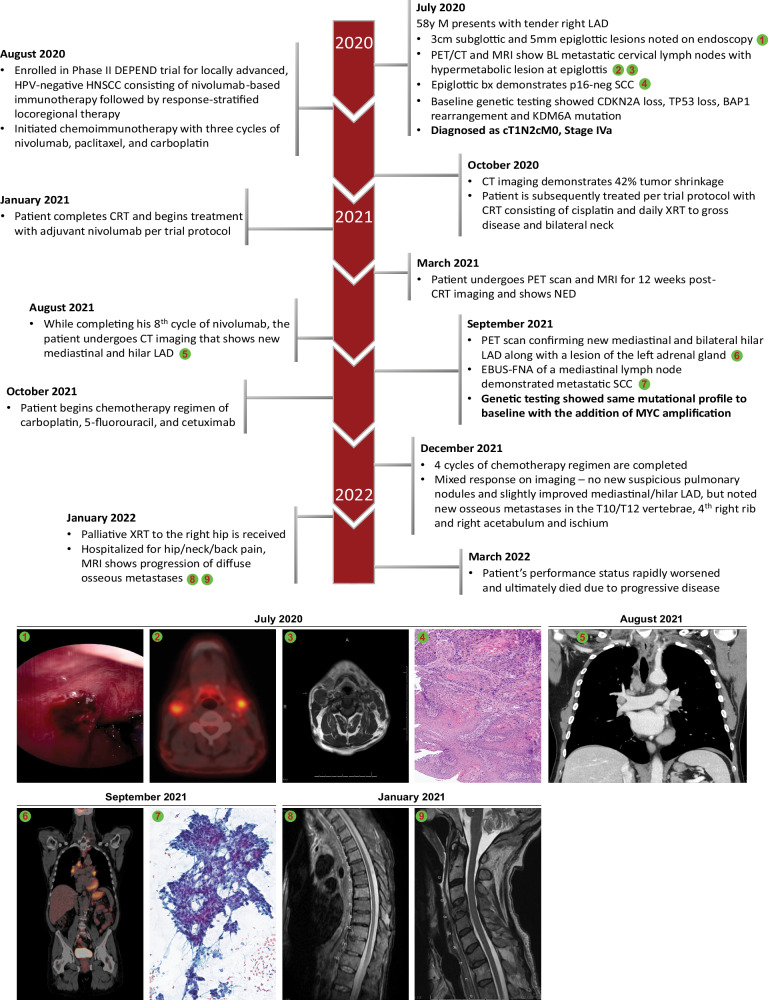
Course of disease. Timeline depicting the course of disease and treatment, during which the patient acquired a *MYC* amplification that was not detected on baseline genetic testing and subsequently developed highly aggressive, immunotherapy-resistant disease. Numbered images at the bottom correspond to the indexes indicated across the course of the disease depicted above. BL: bilateral, EBUS-FNA: endobronchial ultrasound-guided fine-needle aspiration.

A 58-year-old male with an 80-pack-year tobacco history but quit at diagnosis presented in July 2020 noting a right-sided neck mass (Fig. 1). A CT soft tissue neck with contrast demonstrated abnormally enlarged bilateral lymph nodes with a heterogenous appearance and a soft tissue prominence of the anterior hypopharynx at the level of the glottis. A subsequent ultrasound-guided fine-needle aspiration of a right cervical node demonstrated scant metastatic squamous cell carcinoma involving fibrous and lymphoid tissue, at which time the patient was referred to our institutional multidisciplinary head and neck cancer team. Upon endoscopic evaluation, a 3 cm epiglottic pedunculated lesion was noted. A biopsy of the epiglottic lesion was performed and demonstrated squamous cell carcinoma. Immunohistochemical staining for *p16* was negative. Subsequently, a PET/CT scan was conducted, which showed metastatic cervical lymph nodes, most prominent at bilateral level 2, with mild hypermetabolic activity of a lesion along the anterior commissure of the glottis and was staged as (cT1N2cM0, Stage IVa, American Joint Committee on Cancer 8th edition). A baseline Oncoplus molecular analysis was retrospectively conducted on the primary epiglottic specimen, showing *CDKN2A* loss, *TP53* loss, *BAP1* rearrangement, and *KDM6A* mutation. The specimen was found to be microsatellite stable (MSS) and had a tumor mutation burden (TMB) of 18.0 mutations/mb. PD-L1 immunohistochemical staining was negative (CPS < 1%).

The patient was enrolled in a phase II clinical trial for locally advanced, HPV-negative HNSCC evaluating nivolumab-based chemoimmunotherapy followed by response-stratified locoregional therapy (NCT03944915). He initiated induction chemoimmunotherapy with three cycles of nivolumab 360 mg day 1, paclitaxel 100 mg/m2 days 1/8/15, and carboplatin AUC 5 day 1 of 21-day cycle followed by imaging demonstrating 42% tumor shrinkage per RECIST 1.1 and was subsequently treated per protocol with chemoradiotherapy (CRT) consisting of cisplatin 100 mg/m2 q21 days with daily fractionated radiation therapy (RT) at 2 Gy per fraction to total of 70 Gy over 35 fractions to gross disease and bilateral neck. Following completion of CRT he initiated adjuvant nivolumab per protocol. Three months after completion of CRT, the patient underwent PET demonstrating a complete metabolic response. MRI demonstrated no residual cervical lymph node metastases or discernible measurable laryngeal tumor consistent with a complete response by RECIST 1.1 criteria, and direct laryngoscopy showed a complete clinical response. However, during adjuvant nivolumab, six months after completion of chemoradiation, the patient underwent CT imaging demonstrating new mediastinal and hilar lymphadenopathy. A subsequent PET scan showed 18F-fluorodeoxyglucose (FDG) avidity of the mediastinal and bilateral hilar adenopathy along with a left adrenal nodule. Endobronchial ultrasound and fine-needle aspiration of a mediastinal lymph node demonstrated p16 negative, metastatic SCC in September 2021. Repeat Oncoplus molecular analysis was performed on the mediastinal lymph node and revealed an acquired *MYC* amplification in addition to the genetic aberrations detected in the baseline analysis (e.g., *CDKN2A* and *TP53* loss, *BAP1* rearrangement as well as *KDM6A* mutation) supporting a clonal relationship between primary and progressive disease. Of note, this biopsy was also found to be PD-L1 negative on immunohistochemical staining.

The patient started a chemotherapy regimen consisting of carboplatin (AUC 5 day 1), 5-fluorouracil (5-FU, 1000 mg/m2/d days 1–4), and cetuximab (400 mg/m2 loading followed by 250 mg/m2 weekly of 21-day cycle). Repeat imaging demonstrated progressive disease with interval increase in both the intrathoracic lymphadenopathy (LAD) and adrenal lesion as well as new vertebral metastases in T10 and T12 after four cycles of chemotherapy/cetuximab. The patient received palliative XRT to the right hip, however, his performance status rapidly worsened and he ultimately died from progressive disease five months after starting chemotherapy/cetuximab for recurrent/metastatic HNSCC. Given the observation of an acquired *MYC* amplification upon rapidly progressive disease while receiving anti-PD-1 therapy following an initial partial response, we proceeded to characterize clinical-pathologic features of *MYC* amplified HNSCC from our internal dataset, performed RNA-Seq of the paired samples with and without *MYC* amplification from our case study, and analyzed transcriptomic data of *MYC* amplified cases obtained from TCGA-HNSC dataset.

### Clinical-pathologic characterization of recurrent MYC amplified HNSCC

To further interrogate the clinical and molecular profile of tumors with acquired *MYC* amplification, we performed a retrospective medical records review of patients with HNSCC treated at our institution between 2018 and 2021. We identified 8 cases (including the index patient) who were positive for *MYC* amplification based on the OncoPlus assay at the time of recurrence, and 48 matched control cases bearing a wild-type *MYC* (Supplementary Data 1). *MYC* amplified cases demonstrated a median age of 61, 25% were p16 positive oropharynx, all cases (100%) had the local disease at the time of diagnosis and initially received chemoradiotherapy, while the majority (63%) ultimately received immunotherapy during their treatment course. *MYC* amplification was significantly associated with enrichment for the laryngeal primary site (Supplementary Table 1) and showed numerical trend toward elevated frequency of *TP53* and *CDKN2A* genetic aberrations, similar to previous reports (Supplementary Table 2)^35–37^. While median age, gender, treatment modality, p16 status (a surrogate biomarker for HPV-positivity), as well as tumor stage at diagnosis, were not different between the two groups, *MYC* amplified patients showed non-significant trend toward higher rates of developing recurrent and/or metastatic disease following primary therapy, with 100% of cases among *MYC* amplified compared to 72.9% among the wild-type *MYC* counterparts (*p* = 0.08; Supplementary Table 1). Furthermore, although the overall survival was not statistically significantly different with this small sample size available for analysis, median survival of *MYC* amplified patients was 40.6 months, compared to 49.1 months across the *MYC* wild-type individuals (Supplementary Table 1)^36^.

### RNA-Seq analysis of paired patient samples with acquired MYC amplification

**Fig. 2:**
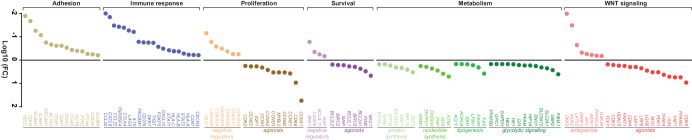
RNA-Seq analysis of rapidly progressive, metastatic disease after the acquisition of a *MYC* amplification. RNA sequencing was performed on the matched tumor specimens collected at baseline and upon progression. The reads were aligned against the human reference genome GRCh38/hg38 and gene counts were calculated. The figure indicates fold change between expression levels of the selected genes in tumor biopsy collected upon disease progression (acquired MYC amplification) and baseline specimen (wild-type *MYC*). Genes with fold change value ± 1.5 were included and grouped by their major roles in WNT signaling, metabolism, survival, and proliferation signaling networks.

Given that transcriptional changes associated with the acquisition of *MYC* amplification in setting of immunotherapeutic resistance is poorly understood in HNSCC, we next performed RNA-Seq analysis of the tumor specimens collected from the patient described in the case report at baseline and upon rapid progression of R/M disease while receiving nivolumab (Supplementary Data 2). A purely descriptive comparative transcriptomic analysis of paired samples showed that genes known to be modulated by *MYC* were upregulated at disease progression, such as WNT/$\beta$-catenin pathway agonists, genes central to the glycolytic pathway (e.g., encoding phosphofructokinase, hexokinase II and enolase), fatty acid metabolism (e.g., acetyl-CoA carboxylase, fatty acid synthase, and ATP-citrate lyase), regulators of nucleotide (e.g., ribonucleotide reductase and ectonucleoside triphosphate diphosphohydrolase) and protein (e.g., eukaryotic translation and elongation factors) synthesis networks, as well as molecules that play role in cell proliferation and survival (Fig. 2). Conversely, WNT antagonists, tumor suppressors known to negatively regulate cell cycle, apoptosis and cell adhesion were downregulated in the *MYC* amplified disease. Notably, genes involved in host anti-tumor immune response such as immune activation regulators, class I human leukocyte antigens (HLAs), STAT 1/2, and chemokines associated with recruitment of immunosuppressive cells were almost universally downregulated in the *MYC* amplified tumor compared to baseline. While these descriptive observations may provide a snapshot of the transcriptomic changes associated with acquired *MYC* amplification, thousands of genes (nearly 15% of transcriptome) are predicted to be direct targets of MYC, highlighting the complexity of the MYC-regulated signaling network^38–40^.

### Acquisition of MYC amplification is associated with broad transcriptomic changes

**Fig. 3:**
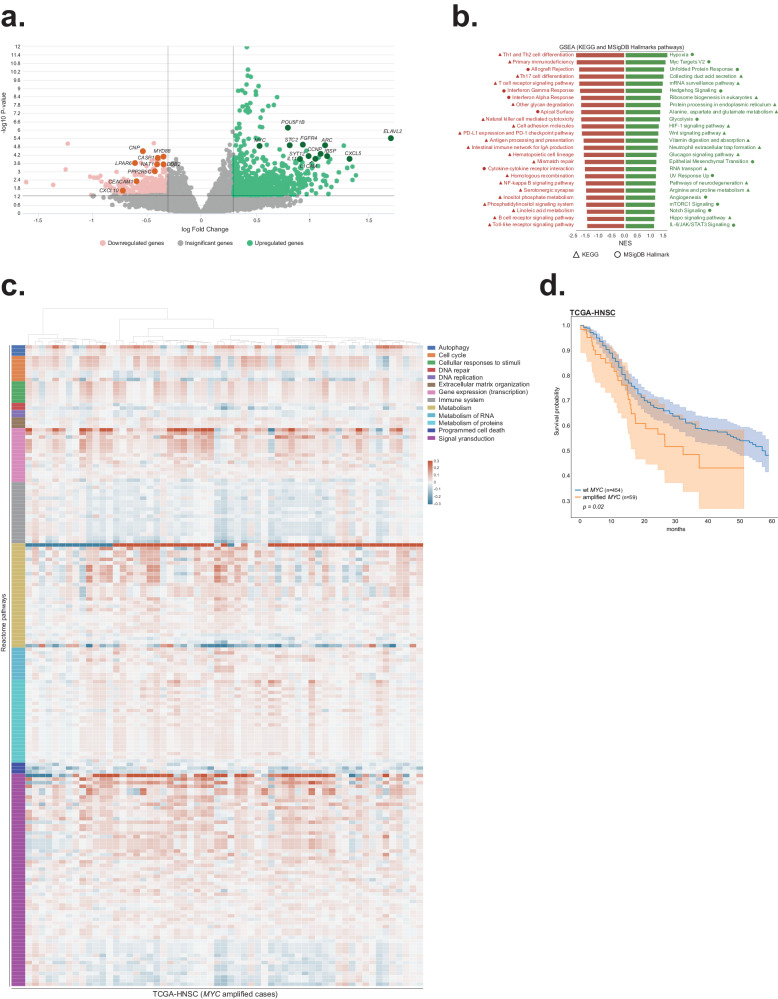
Acquisition of MYC amplification is associated with broad transcriptomic changes. **a** Volcano plot based on the TCGA-HNSC transcriptomic data comparing patients carrying amplified and wild-type *MYC*. Logarithmic fold-changes and FDR-corrected *q*-values were used to build volcano plot for differentially expressed genes (Q-value < 0.05). Green and pink colors depict significantly up-regulated and down-regulated genes respectively. Darker colors highlight genes known to be associated with HNSCC carcinogenesis. **b** Gene set enrichment analysis based on the TCGA-HNSC transcriptomic data comparing patients carrying amplified and wild-type *MYC*. Analysis was performed using two collections of gene sets obtained from the Enrichr library: MSigDB Hallmark 2020 and KEGG 2021 Human. Top 25 upregulated (green) and down-regulated (red) pathways from MSigDB Hallmark gene sets (circle) and KEGG database (triangle) are shown. All indicated pathways have FDR *q*-val < 0.05. **c** Pathway activation heatmap comparing TCGA-HNSC tumors with amplified and wild-type *MYC*. Pathway activation (shades of red) or inhibition (shades of blue) is inferred based on the scores obtained from InSilico Pathway Activation Network Decomposition Analysis (iPANDA) algorithm applied to the Reactome pathways database. Pathways are grouped according to the Reactome’s “superpathways” that describe normal cellular functions. **d** Kaplan–Meier survival probabilities with 95% CIs for patients with *MYC* amplification and wild-type MYC tumors (two-sided log-rank test *p* = 0.02).

As we could not generate statistically meaningful conclusions based on the analysis of a single case sequencing, we next performed transcriptomic evaluation using samples obtained from the TCGA-HNSC dataset (Supplementary Data 3). Notably, tumors carrying amplified *MYC* (*n* = 59) were enriched for laryngeal disease, *TP53* and *CDKN2A* mutations (Supplementary Table 3, Supplementary Fig. 1), and exhibited significant upregulation of numerous driver genes known to be associated with carcinogenesis in several types of cancer, including HNSCC (e.g. *ELAVL2*, *CXCL5*, *FGFR4*, *CCNP*, *STC2*, *POU5F1B* and *SYT12*)^41–50^. Significantly down-regulated genes contained known tumor suppressors and regulators of immune response (*MYD88*, *NAT1*, *DDB2*, *PPP2R5C*, *LPAR6*, *CNP*, *CASP1, CXCL10, and CEACAM1*)^51–66^ (Fig. 3a, Supplementary Data 4). Interestingly, the relative expression of T cell recruiting chemokines, immune checkpoints, genes associated with antigen presentation, and IFN-γ signaling showed a trend toward downregulation in *MYC* amplified tumors (Supplementary Fig. 2). As analysis of the expression patterns fails to capture subtle differences between samples that arise from dynamic interactions between genes at the signaling level^67^, we next applied pathway-based scoring methods that project gene expression data into the biological networks. Gene set enrichment analysis (GSEA)^68^ using KEGG and MSigDB Hallmark pathway databases revealed that the top up-regulated pathways in *MYC* amplified tumors were represented by processes that promote tumor growth, metastasis, and chemoresistance such as hypoxia, protein metabolism, glycolysis, epithelial-mesenchymal transition, angiogenesis, WNT (which has been shown to transcriptionally activate *MYC* expression)^69^, NOTCH, mTORC1, Hedgehog, Hippo and IL-6/JAK/STAT3 signaling (Fig. 3b, Supplementary Data 5). On the other hand, pathways associated with anti-tumor immune response (e.g., interferon-gamma response, T cell receptor signaling, natural killer cell-mediated cytotoxicity, antigen processing and presentation, Th1/Th2 cell differentiation, and toll-like receptor signaling) were enriched among the top down-regulated signaling axes (Fig. 3b), which may explain, in part, the poor effectiveness of immunotherapies against MYC-driven malignancies^70^. To query the transcriptomic data in more detail, iPANDA algorithm was pursued to predict differential activation of pathways retrieved from the Reactome database, which provides the hierarchical organization of signaling axes grouped into broader domains of biological functions^67,71^. Supporting the GSEA analysis, PandaOmics revealed that signaling networks associated with cell cycle progression, gene expression, signal transduction, and metabolism were upregulated in patients carrying the *MYC* amplification, whereas cellular processes related to the immune system and apoptosis were downregulated (Fig. 3c, Supplementary Data 6). Notably, while HPV frequency, age, gender, and tumor stage at diagnosis were not different between the two groups (Supplementary Table 3), the 5-year overall survival of patients with *MYC* amplified tumors was lower compared to the *MYC* wild-type counterparts (Fig. 3d), supporting the relevance of the molecular landscape changes described above to patient’s outcomes.

## Discussion

Immune checkpoint inhibition alone or in combination with chemotherapy has an established role in R/M HNSCC and is associated with improved survival, yet mechanisms of acquired resistance are poorly understood^5–9^. Here we present a unique case of a patient with *p16* negative SCC of the epiglottis, staged as T1N2cM0 (IVA), who had a partial response following induction nivolumab/chemotherapy followed by definitive concurrent CRT, demonstrating a complete metabolic response. Interestingly, despite early response to therapy, this patient unfortunately developed rapidly progressive metastatic disease while actively receiving adjuvant nivolumab and was found to have acquired a *MYC* amplification that was not present on his baseline biopsy. This led to the question of how the acquisition of *MYC* amplification in this patient’s rapidly progressive metastatic HNSCC may contribute to molecular pathway dysfunction that promoted immune evasion and therapeutic resistance. While *MYC* amplification is well described in epithelial-type malignancies and associated with a poor prognosis^25,72,73^, its role in HNSCC therapeutic resistance and immune evasion has not been characterized.

We initially queried our internal database for HNSCC patients with *MYC* amplification at the time of recurrence along with *MYC* wild-type controls to characterize the clinicopathologic features of this cohort. Interestingly, we found that *MYC* amplified HNSCCs were enriched for larynx primary site, and appeared to be associated with higher frequencies of *TP53* and *CDKN2A* genetic aberrations and HPV-negative disease, consistent with previous reports^35,74^. Yet despite these findings, *MYC* amplification in our cohort was also identified among other subsites and among patients with HPV-positive HNSCC. Although statistically significant prognostic comparisons were limited by the small sample size in our internal cohort and *MYC* status was only evaluable at the time of recurrence, there did appear to be a numerical trend towards worse median survival with *MYC* amplification, as well as higher rates of recurrence, despite the majority of patients ultimately receiving immunotherapy in addition to chemoradiotherapy, conforming with previous observations^36^.

Although these findings were suggestive of unique clinicopathologic patterns and signal of prognostic implications, the potential role of acquired *MYC* alterations in therapeutic resistance including immune evasion in HNSCC could not be interrogated. Therefore, we performed *RNA-Seq* analysis on paired patient samples from our index case to compare in descriptive terms baseline *MYC* wild-type tumor sample, with acquired *MYC* amplification at the time of distant metastatic disease while receiving anti-PD-1 therapy. Notably, we found relative upregulation of genes that play role in WNT/$\beta$-catenin signaling (which is strongly correlated with immune exclusion across multiple human cancer types)^75^, the glycolytic pathway, fatty acid metabolism, nucleotide regulators, and protein synthesis networks. At the same time, we identified relative downregulation of tumor suppressors known to negatively regulate cell cycle, apoptosis, and cell adhesion, consistent with previous suggestions that MYC amplification in HNSCC is associated with activation of DNA repair pathways and promoting DNA damage checkpoint response, which may contribute to chemotherapeutic and radiotherapeutic resistance^76,77^. Interestingly, paired RNA-Seq analysis in our patient also identified relative downregulation of genes involved in host anti-tumor immune response including immune activation regulators, class I HLAs, STAT 1/2, and chemokines associated with recruitment of immunosuppressive cells within the tumor microenvironment. Previous studies have corroborated these findings suggesting that *MYC* activation leads to the enrichment of immunosuppressive tumor microenvironment such as regulatory T-cells, along with the exclusion of CD8+ T-cells and NK cells, in several tumor types including HNSCC, and supported by reports of differential metastasis response to immune checkpoint inhibitors with *MYC* amplification^31,36,70^. While formal statistical comparisons cannot be performed in a single patient, these findings suggest potential *MYC*-mediated mechanisms of immune evasion, that may explain the development of secondary resistance to immune checkpoint inhibitors with the acquisition of a *MYC* amplification in this patient. Of note, this patient did not have PD-L1 expression in his tumor at baseline, which may also explain immune evasion despite immune checkpoint inhibitor therapy in this patient. However, a very high tumor mutational burden at baseline (18 mut/MB) was noted which would be anticipated to predict response to immune checkpoint inhibition even without PD-L1 expression via generation of higher neoantigen burden facilitating deeper and more durable responses with immune checkpoint inhibition^78^. This supports our hypothesis that alternative acquired *MYC*-driven immunosuppressive processes may be playing a role in this patient’s rapid disease progression while receiving nivolumab, even if robust neoantigen is present^79,80^.

Given our inability to generate statistically meaningful conclusions based on a single case paired sequencing analysis, we interrogated the transcriptomic changes within the publicly available TCGA-HNSC dataset. We again identified enrichment for laryngeal disease and HPV-negative patients, along with increased association with *TP53* (as has been characterized previously in the literature and our internal dataset), and a statistically significant worsening of survival compared with *MYC* wild-type^35,74^. As negative HPV status and the presence of TP53 mutations are independent predictors of an aggressive disease phenotype in HNSCC^81,82^, they may also underlie the inferior overall survival seen in MYC amplified patients, requiring mechanistic investigation. Our TCGA-HNSC analysis similarly found upregulation in carcinogenesis driver genes, along with downregulation of tumor suppressor and immune response regulatory pathways. Furthermore, iPANDA pathway activation prediction analysis^67,83,84^ specifically identified upregulation of signaling networks associated with cell cycle progression, signal transduction, and metabolism, while immune cellular processes and apoptosis were indeed downregulated. These provide additional support for the hypothesis, driven from *RNA-Seq* analysis of a single case, for *MYC*-driven mechanisms of immune evasion playing a key role in addition to chemoradiotherapy resistance in the poor outcome for this patient despite initial response to immunotherapy-based multimodality treatment. Our findings highlight the need for more work interrogating *MYC*-mediated mechanisms of acquired resistance to immunotherapy in HNSCC, as well as potential combinatorial therapeutic strategies to target the cellular processes that drive this therapeutic resistance^24,33,34^.

Limitations to our study include the single patient case which limits definitive conclusions, as well as the small number of *MYC* amplified cases in our internal cohort limiting power for statistical comparisons. Additionally, there may be selection bias within a single institution for DNA sequencing and potential confounders due to the retrospective nature of comparisons of *MYC* amplified with *MYC* wild-type cohorts which limit definitive conclusions and generalizability. Furthermore, transcriptomic data for TCGA-HNSC patients was obtained from primary tumors, which may not fully reflect the complex network of MYC-induced signaling aberrations that occur upon progression of R/M disease. Finally, while our transcriptomic pathway analysis focused on potential *MYC*-driven mechanisms of immune evasion, there is likely therapeutic resistance to chemotherapy, radiotherapy, and targeted therapy also at play. Further work is needed to mechanistically differentiate therapeutic resistance to immunotherapy and other therapeutic treatment modalities. As there is no accepted HNSCC cellular or animal model mimicking MYC amplification, we are currently creating a bank of HNSCC organoids carrying amplified MYC. The mechanistic studies using these models will be report in a follow-up publication.

In conclusion, our study suggests that *MYC* amplification in HNSCC, while a relatively uncommon occurrence, may play a key role not only in chemo and radioresistance but suggests that acquired clonal alteration may drive resistance to immunotherapy through a variety of potential immune suppressive mechanisms, warranting further study. Characterization of potential mechanisms of *MYC*-driven immune evasion in HNSCC could lead to the development of biomarker-selected targeted combinatorial therapeutic strategies to overcome resistance to immunotherapy.

## Methods

### Case report/series

A single patient treated at the University of Chicago Medical Center (UCMC) on a prospective clinical trial (NCT03944915) was selected for a case analysis based on the identification of an acquired *MYC* amplification noted on the UCMC in-house OncoPlus next-generation sequencing-based genomic sequencing platform (https://uchicagomedlabs.testcatalog.org/show/NGPLSF)^85^. The prospective “DEPEND” trial was a single-arm phase II study of nivolumab, paclitaxel, and carboplatin, followed by response-adaptive chemoradiation and adjuvant nivolumab for patients with locoregionally advanced HPV-negative HNSCC as described previously^86^.

A patient signed and informed consent under the University of Chicago Institutional Review Board (IRB) approval and provided consent to publish on the basis of anonymized data. Seven additional HNSCC patients with *MYC* amplification and 48 patients with wild-type *MYC* to serve as controls were identified from the OncoPlus genetic screen database^85^. There were no *MYC* amplified HNSCC patients excluded from the analysis. A retrospective chart review was conducted following IRB-approved protocols to collect demographic and clinical data (including disease course, treatments, and survival). The present study was conducted in accordance with all relevant ethical regulations including the Declaration of Helsinki.

### RNA sequencing

Tumor samples were obtained following IRB-approved protocols. Informed written consent was obtained from the patient before sampling. Paraffin-embedded slides were microdissected to obtain >60% neoplastic cells. Neoplastic cellularity was estimated from the sequential slides, which highly reflect cellularity of the section used for RNA sequencing. Total RNA was isolated with AllPrep DNA/RNA Kit (Qiagen) and measured using Agilent 2100 Bioanalyzer. Libraries were prepared using the TruSeq RNA Library Prep Kit and sequencing was performed using the NovaSeq platform (Illumina). The quality control assessment of raw paired-end sequencing reads was performed by FastQC^87^. Further, the reads were aligned against the human reference genome GRCh38/hg38, and read counts per gene was calculated using STAR (v2.6.1d)^88^. Visualization for Fig. 2 was generated using GraphPad Prism (version 10.1.1).

### Transcriptomic analysis of the data obtained from TCGA-HNSC cohort

Raw counts pre-processing included Upper-quartile normalization and log2-transformation. Patients were segregated into *MYC* amplified and *MYC* wild-type subgroups based on GISTIC2 call. Specifically, TCGA-HNSC thresholded gene-level copy number variation (CNV) was estimated using the GISTIC2 method^89^. Copy number profile was measured experimentally using whole genome microarray at a TCGA genome characterization center. Subsequently, GISTIC2 method was applied using the TCGA FIREHOSE pipeline to produce gene-level copy number estimates. GISTIC2 further thresholded the estimated values to −2,−1,0,1,2, representing homozygous deletion, single copy deletion, diploid normal copy, low-level copy number amplification, or high-level copy number amplification. The high-level thresholds are calculated on a sample-by-sample basis and are based on the maximum median arm-level amplification copy number found in the sample. Only samples with GISTIC2 value of 2 (high-level copy number amplification) were considered MYC amplified (Supplementary Data 3). Gene expression data were uploaded into PandaOmics, an AI-driven target discovery platform, for subsequent analysis. Differential gene expression analysis has been performed using the limma-voom package inside the PandaOmics platform^90^. Obtained gene-wise *p*-values were corrected by the Benjamini–Hochberg procedure. Logarithmic fold-changes (LFC) and FDR-corrected Q-values were used to build volcano plot for differentially expressed genes (DEGs) (*Q*-value < 0.05) (Supplementary Data 4). Then, each of the DEGs was given a status of being oncogene and(or) tumor suppressor gene following the mappings mined from the OncoKB database^91,92^. To study the dysregulation of cellular processes between *MYC* amplified and *MYC* wild-type cases, iPANDA algorithm^67^ was applied using the Reactome pathways database^93^. iPANDA calculates the activation or inhibition score for each pathway by combining precalculated gene coexpression data with gene importance factors based on the degree of differential gene expression and pathway topology decomposition. Significantly dysregulated pathways with iPANDA score > 0.01 or < −0.01 were considered as activated and inhibited, respectively (Supplementary Data 6). *P*-values for the iPANDA pathway activation scores were obtained using weighted Fisher’s combined probability test. Detailed description and statistical credibility of the iPANDA score was previously published^67^. Unsupervised complete-linkage clustering was performed following Farthest Point algorithm and Euclidean metric^94^. Gene set enrichment analysis (GSEA) was performed with the GSEAPY python package^95^ using two collections of gene sets obtained from Enrichr library^96^ - MSigDB_Hallmark_2020 and KEGG_2021_Human (Supplementary Data 5).

### Statistical analysis

Survival analysis was prepared in PandaOmics using the Kaplan–MeierFitter function from the lifelines Python package (two-sided log-rank test). The differences in clinical and disease-specific characteristics i(e.g. age, gender, primary site, HPV/p16 status, TNM stage, gene mutation frequency, etc.) between *MYC* amplified and wild-type cases (in the internal cohort and TCGA-HNSC dataset) were calculated by two-sample *t*-tests and chi-square tests of independence using GraphPad Prism software. The significance level was defined as 0.05.

### Reporting summary

Further information on research design is available in the Nature Research Reporting Summary linked to this article.

### Supplementary information

Reporting summary

Supplementary Material

Supplementary Data 1

Supplementary Data 2

Supplementary Data 3

Supplementary Data 4

Supplementary Data 5

Supplementary Data 6

## Data Availability

The authors declare that the data supporting the findings of this study are available within the article and its supplementary information files. Source data are provided with this paper. Patients’ de-identified data (such as diagnosis, gender, averaged age, and treatment type) is provided in the manuscript.

## Code availability

No code was developed in this study. GSEA software is freely available for download at https://www.gsea-msigdb.org/gsea/index.jsp. PandaOmics is industry-grade commercial software platform used since 2020. The platform is available at https://pandaomics.com. A trial access to the platform is available from InSilico Medicine upon request. A workflow for running the platforms is described in the “Methods” section.
