# Supplementary material for: Acquired resistance to immunotherapy and chemoradiation in MYC amplified head and neck cancer
Source: NPJ Precis Oncol. 2024 May 23;8:114. doi: 10.1038/s41698-024-00606-w (PMC11116544; doi:10.1038/s41698-024-00606-w)
Supplement: Supplementary file 1 — Reporting summary [file 41698_2024_606_MOESM1_ESM.pdf]

nature portfolio

Corresponding author(s): Evgeny Izumchenko M.Sc., Ph.D.
Ari Rosenberg M.D.

Last updated by author(s): Feb 22, 2024

Reporting Summary

Nature Portfolio wishes to improve the reproducibility of the work that we publish. This form provides structure for consistency and transparency in reporting. For further information on Nature Portfolio policies, see our Editorial Policies and the Editorial Policy Checklist.

Statistics

For all statistical analyses, confirm that the following items are present in the figure legend, table legend, main text, or Methods section.

n/a	Confirmed	
☐	☒	The exact sample size (*n*) for each experimental group/condition, given as a discrete number and unit of measurement
☒	☐	A statement on whether measurements were taken from distinct samples or whether the same sample was measured repeatedly
☐	☒	The statistical test(s) used AND whether they are one- or two-sided<br>*Only common tests should be described solely by name; describe more complex techniques in the Methods section.*
☐	☒	A description of all covariates tested
☒	☐	A description of any assumptions or corrections, such as tests of normality and adjustment for multiple comparisons
☐	☒	A full description of the statistical parameters including central tendency (e.g. means) or other basic estimates (e.g. regression coefficient) AND variation (e.g. standard deviation) or associated estimates of uncertainty (e.g. confidence intervals)
☐	☒	For null hypothesis testing, the test statistic (e.g. *F*, *t*, *r*) with confidence intervals, effect sizes, degrees of freedom and *P* value noted<br>*Give P values as exact values whenever suitable.*
☒	☐	For Bayesian analysis, information on the choice of priors and Markov chain Monte Carlo settings
☒	☐	For hierarchical and complex designs, identification of the appropriate level for tests and full reporting of outcomes
☒	☐	Estimates of effect sizes (e.g. Cohen's *d*, Pearson's *r*), indicating how they were calculated

Our web collection on statistics for biologists contains articles on many of the points above.

Software and code

Policy information about availability of computer code

Data collection	No software was used for data collection
Data analysis	Gene set enrichment analysis (GSEA) was performed with the GSEAPY python package.<br>To study the dysregulation of cellular processes between MYC amplified and MYC wild-type cases, iPANDA algorithm (InSilico Medicine) was applied using the Reactome pathways database.<br>Differential gene expression analysis has been performed using the limma-voom package inside the PandaOmics platform (InSilico Medicine).

For manuscripts utilizing custom algorithms or software that are central to the research but not yet described in published literature, software must be made available to editors and reviewers. We strongly encourage code deposition in a community repository (e.g. GitHub). See the Nature Portfolio guidelines for submitting code & software for further information.

Data

Policy information about availability of data

All manuscripts must include a data availability statement. This statement should provide the following information, where applicable:

- Accession codes, unique identifiers, or web links for publicly available datasets
- A description of any restrictions on data availability
- For clinical datasets or third party data, please ensure that the statement adheres to our policy

The authors declare that the data supporting the findings of this study are available within the article and its supplementary information files. Source data are provided with this paper.

Research involving human participants, their data, or biological material

Policy information about studies with human participants or human data. See also policy information about sex, gender (identity/presentation), and sexual orientation and race, ethnicity and racism.

Reporting on sex and gender	Data collected from both men and women over age of 18 were included for the analysis, and there was no preferential selection requirement by gender.
Reporting on race, ethnicity, or other socially relevant groupings	Data collected from patients regardless of race or ethnicity were eligible to be used in this study.
Population characteristics	Data from patients with head and neck cancer from internal database and TCGA-HNSC was used for the analysis. Demographic and clinicopathological characteristics of the MYC amplified and MYC wild-type patients obtained from TCGA-HNSC dataset is summarized in Supp. Table 9. Demographic and clinical characteristics of the 8 MYC amplified and 48 wild-type patients treated at the University of Chicago is summarized in Supp. Table 5.
Recruitment	Only retrospective data was used for the analysis. No patients were requited for this study.
Ethics oversight	Data from 56 patients treated at the University of Chicago Medical Center on a prospective clinical trial (NCT03944915) was selected for a case analysis. Trial was approved by IRB and registered on clinicaltrials.gov (https://classic.clinicaltrials.gov/ct2/show/NCT03944915). The remaining data was obtained from TCGA.

Note that full information on the approval of the study protocol must also be provided in the manuscript.

Field-specific reporting

Please select the one below that is the best fit for your research. If you are not sure, read the appropriate sections before making your selection.

☒ Life sciences ☐ Behavioural & social sciences ☐ Ecological, evolutionary & environmental sciences

For a reference copy of the document with all sections, see nature.com/documents/nr-reporting-summary-flat.pdf

Life sciences study design

All studies must disclose on these points even when the disclosure is negative.

Sample size	We used all the TCGA-HNSC data available. No sample size calculation was needed.
Data exclusions	No data was excluded from the analysis.
Replication	As this is a summary analysis of existing data, no experimental replication was used in the study.
Randomization	Patients were allocated to experimental groups based on the presence of MYC amplification.
Blinding	Internal data was collected from an open-label study. TCGA-HNSC data is publicly available. No blinding procedures were used.

Reporting for specific materials, systems and methods

We require information from authors about some types of materials, experimental systems and methods used in many studies. Here, indicate whether each material, system or method listed is relevant to your study. If you are not sure if a list item applies to your research, read the appropriate section before selecting a response.

Materials & experimental systems

n/a	Involved in the study	
☐	☐	Antibodies
☒	☐	Eukaryotic cell lines
☒	☐	Palaeontology and archaeology
☒	☐	Animals and other organisms
☐	☒	Clinical data
☒	☐	Dual use research of concern
☒	☐	Plants

Methods

n/a	Involved in the study	
☒	☐	ChIP-seq
☒	☐	Flow cytometry
☒	☐	MRI-based neuroimaging

Antibodies

Antibodies used	NA
Validation	NA

Clinical data

Policy information about clinical studies

All manuscripts should comply with the ICMJE guidelines for publication of clinical research and a completed CONSORT checklist must be included with all submissions.

Clinical trial registration	NCT03944915
Study protocol	This manuscript is not a clinical study. Details of the study which was used as a source of patients data can be found at https://classic.clinicaltrials.gov/ct2/show/NCT03944915
Data collection	University of Chicago. Start Date : August 26, 2019. The trial is ongoing.
Outcomes	Primary Outcome Measures and Secondary Outcome Measures can be found at: https://classic.clinicaltrials.gov/ct2/show/NCT03944915

Plants

Seed stocks	*Report on the source of all seed stocks or other plant material used. If applicable, state the seed stock centre and catalogue number. If plant specimens were collected from the field, describe the collection location, date and sampling procedures.*
Novel plant genotypes	*Describe the methods by which all novel plant genotypes were produced. This includes those generated by transgenic approaches, gene editing, chemical/radiation-based mutagenesis and hybridization. For transgenic lines, describe the transformation method, the number of independent lines analyzed and the generation upon which experiments were performed. For gene-edited lines, describe the editor used, the endogenous sequence targeted for editing, the targeting guide RNA sequence (if applicable) and how the editor was applied.*
Authentication	*Describe any authentication procedures for each seed stock used or novel genotype generated. Describe any experiments used to assess the effect of a mutation and, where applicable, how potential secondary effects (e.g. second site T-DNA insertions, mosiacism, off-target gene editing) were examined.*
