# Supplementary material for: Acquired resistance to immunotherapy and chemoradiation in MYC amplified head and neck cancer
Source: NPJ Precis Oncol. 2024 May 23;8:114. doi: 10.1038/s41698-024-00606-w (PMC11116544; doi:10.1038/s41698-024-00606-w)
Supplement: Supplementary file 2 — Supplementary Material [file 41698_2024_606_MOESM2_ESM.pdf]

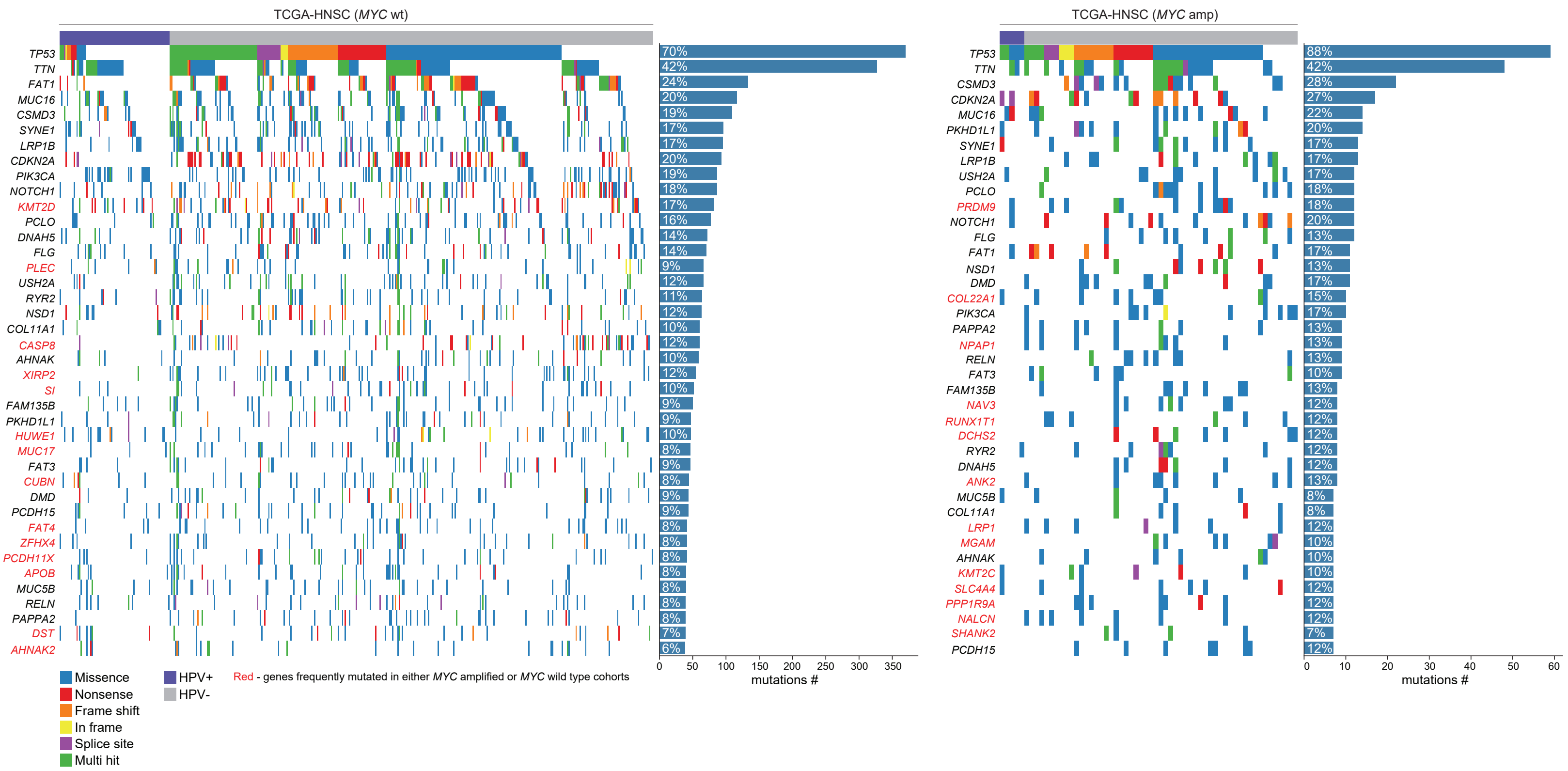

Supplementary Figure 1. CoMut plots was used to visualize the mutational landscape in *MYC* amplified (right) and *MYC* wild-type (left) tumors derived from TCGA-HNSC cohort, and stratified by HPV status. Non-coding and silent variants were excluded. Top 40 most frequently mutated genes are shown.

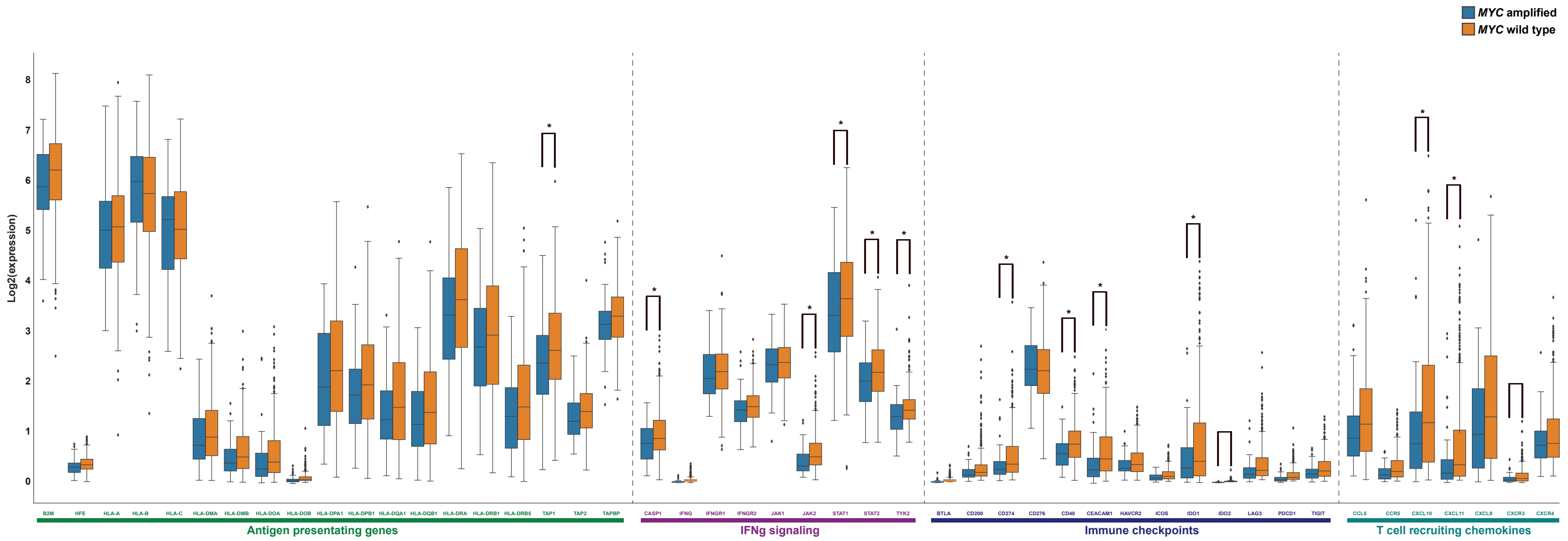

Supplementary Figure 2. The chart indicates relative mRNA expression of genes encoding for T cell recruiting chemokines, immune checkpoints, genes associated with antigen presentation and IFN-γ signaling in TCGA-HNSC tumors with *MYC* amplification (blue boxes) and wild-type *MYC* (orange boxes). Asterisks indicate significant differentially expressed genes between the two cohorts. Box plot indicates the median (solid line), interquartile range (box), and values within 1.5 times the interquartile range (whiskers).

Supplementary Table 1. Comparison of the clinicopathological features and survival of *MYC* amplified and *MYC* wild-type patients treated at the University of Chicago.

	**Amplified MYC (N=8)**	**Wildtype MYC (N=48)**	***P* value**
Age (mean, SD)	61.1 (9.9)	59.0 (11.4)	0.62
Gender (M/F)	5/3	37/11	0.32
Site of primay (n, %)			
Oral Cavity	1 (12.5)	14 (29.1)	0.32
Larynx	3 (37.5)	5 (10.4)	0.04
Hypopharynx	2 (25.0)	3 (6.25)	0.01
Tonsil/Oropharynx	2 (25.0)	26 (54.1)	0.12
p16 positivity (n, %)	2 (25)	20 (42.6)	0.37
TNM staging (n, %)			
Stage I	1 (12.5)	10 (20.8)	0.60
Stage II	6 (75.0)	18 (37.5)	0.05
Stage III	1 (12.5)	9 (18.7)	0.67
Stage IV	1 (12.5)	9 (18.7)	0.67
Tx	0 (0)	2(4.2)	
Treatment modality (n, %)			
Chemotherapy	8 (100)	45 (93.7)	0.46
Radiation	8 (100)	45 (93.7)	0.46
Immunotherapy	5 (62.5)	22 (45.8)	0.38
Targeted Therapy	6 (75)	11 (22.9)	0.01
Surgery	5 (62.5)	23 (47.9)	0.44
Recurrent disease (n, %)	8 (100)	35 (72.9)	0.08
Site of recurrence (n, %)			
Local	3 (37.5)	11 (31.4)	0.30
Distant	3 (37.5)	12 (34.2)	0.45
Both	2 (25)	12 (34.2)	0.99
Median survival (months)	40.6	49.1	0.60

Supplementary Table 2. Most frequently mutated genes in *MYC* amplified and *MYC* wild-type patients treated at the University of Chicago (based on the OncoPlus assay).

	Amplified MYC (N=8)	Wildtype MYC (N=48)
Pathogenic mutation (gene, %, # of patients)		
	TP53 (75%, 6)	TP53 (64.6%, 31)
	CDKN2A (62.5%, 5)	TERT (29.2%, 14)
	CCND1 (25%, 2)	APC (25%, 12)
	KDM6A (25%, 2)	CDKN2A (22.9%, 11)
	PIK3CA (25%, 2)	FAT3 (18.8%, 9)

Supplementary Table 3. Demographic and clinicopathological characteristics of the *MYC* amplified and *MYC* wild-type patients obtained from TCGA-HNSC dataset.

	**ampMYC**	**wtMYC**	**Significance**
HPV pos	5.10%	8.0%	NS
Male	73.3%	78.0%	NS
Female	26.7%	22.0%	
Stage I	57.6%	53.9%	NS
Stage II	20.3%	20.3%	NS
Stage III	11.9%	19.6%	NS
Stage IV	5.1%	3.7%	NS
NA	5.1%	2.4%	NS
Age	61.9	60.9	NS
Current smoker	45.8%	32.4%	0.04
Former smoker	39.0%	41.4%	NS
Never smoker	15.2%	24.0%	NS
Oral Cavity	52.5%	61.4%	NS
Larynx	33.9%	20.9%	0.03
Hypopharynx	1.7%	2.0%	NS
Tonsil/Oropharynx	11.8%	15.6%	NS
TP53	88.2%	70.3%	0.01
CDKN2A	27.0%	20.0%	NS

NS - not significant
